# MetaboDirect: an analytical pipeline for the processing of FT-ICR MS-based metabolomic data

**DOI:** 10.1186/s40168-023-01476-3

**Published:** 2023-02-17

**Authors:** Christian Ayala-Ortiz, Nathalia Graf-Grachet, Viviana Freire-Zapata, Jane Fudyma, Gina Hildebrand, Roya AminiTabrizi, Cristina Howard-Varona, Yuri E. Corilo, Nancy Hess, Melissa B. Duhaime, Matthew B. Sullivan, Malak M. Tfaily

**Affiliations:** 1 Department of Environmental, Science, University of Arizona, Tucson, AZ 85721 USA; 2 Present address: Roche, Pleasanton, CA 94588 USA; 3 Present address: University of California, Davis|Department of Plant Pathology, Davis, CA 95616-8751 USA; 4 Present address: University of Chicago Biological Sciences Division, Metabolomics Platform, Chicago, IL 60637 USA; 5 Department of Microbiology, Ohio State University, Columbus, OH 43210 USA; 6 Center of Microbiome Science, Ohio State University, Columbus, OH 43210 USA; 7 Environmental Molecular Sciences Laboratory, Pacific Northwest National Laboratory, Richland, WA 99354 USA; 8 Department of Ecology and Evolutionary Biology, University of Michigan, Ann Arbor, MI 48109 USA; 9 Department of Civil, Environmental, and Geodetic Engineering, Ohio State University, Columbus, OH 43210 USA

**Keywords:** Metabolites, Organic matter, FT-ICR MS, Biochemical networks

## Abstract

**Background:**

Microbiomes are now recognized as the main drivers of ecosystem function ranging from the oceans and soils to humans and bioreactors. However, a grand challenge in microbiome science is to characterize and quantify the chemical currencies of organic matter (i.e., metabolites) that microbes respond to and alter. Critical to this has been the development of Fourier transform ion cyclotron resonance mass spectrometry (FT-ICR MS), which has drastically increased molecular characterization of complex organic matter samples, but challenges users with hundreds of millions of data points where readily available, user-friendly, and customizable software tools are lacking.

**Results:**

Here, we build on years of analytical experience with diverse sample types to develop MetaboDirect, an open-source, command-line-based pipeline for the analysis (e.g., chemodiversity analysis, multivariate statistics), visualization (e.g., Van Krevelen diagrams, elemental and molecular class composition plots), and presentation of direct injection high-resolution FT-ICR MS data sets after molecular formula assignment has been performed. When compared to other available FT-ICR MS software, MetaboDirect is superior in that it requires a single line of code to launch a fully automated framework for the generation and visualization of a wide range of plots, with minimal coding experience required. Among the tools evaluated, MetaboDirect is also uniquely able to automatically generate biochemical transformation networks (*ab initio*) based on mass differences (mass difference network-based approach) that provide an experimental assessment of metabolite connections within a given sample or a complex metabolic system, thereby providing important information about the nature of the samples and the set of microbial reactions or pathways that gave rise to them. Finally, for more experienced users, MetaboDirect allows users to customize plots, outputs, and analyses.

**Conclusion:**

Application of MetaboDirect to FT-ICR MS-based metabolomic data sets from a marine phage-bacterial infection experiment and a *Sphagnum* leachate microbiome incubation experiment showcase the exploration capabilities of the pipeline that will enable the research community to evaluate and interpret their data in greater depth and in less time. It will further advance our knowledge of how microbial communities influence and are influenced by the chemical makeup of the surrounding system. The source code and User’s guide of MetaboDirect are freely available through (https://github.com/Coayala/MetaboDirect) and (https://metabodirect.readthedocs.io/en/latest/), respectively.

Video Abstract

**Supplementary Information:**

The online version contains supplementary material available at 10.1186/s40168-023-01476-3.

## Background

Microorganisms play crucial roles in a host of fundamental ecological processes and are needed for maintaining a healthy global ecosystem [1, 2]. They are responsible for the mobilization, transformation, and storage of natural organic matter (NOM)—the complex mixture of organic compounds present within any system—thus driving the cycling of elements essential for life (e.g., carbon, nitrogen, sulfur) [2–5]. Microorganisms further contribute to the NOM pool, especially the dissolved organic matter (DOM) pool, in both aquatic and terrestrial systems [3, 6]. Environmental conditions such as temperature, and water availability can strongly influence the microbial community structure and function and thus its interaction with NOM [7, 8].

Microorganisms are capable of directly assimilating low molecular weight DOM (< 600 Da) for their metabolic processes, while also producing microbial-derived products and residues that become integral components of NOM such as proteins, polysaccharides, and cell wall polymers [9]. The quantity and the quality of OM in a given ecosystem are dependent on its microbiome composition and the environmental conditions present in that system. They are also dependent on several microbial regulatory processes, such as transcription, translation, protein interactions, and their interactions with the biotic and abiotic components of the system [10–14]. Characterizing OM molecular composition is therefore vital for understanding the role that microorganisms play in all major element biogeochemical cycles and can constitute an important predictor of the response of the biological systems to environmental perturbations [8, 15].

Natural organic matter is a complex mixture of organic compounds whose size, and other molecular properties are perceived as a continuum [16, 17]. Because separating and analyzing each component of the NOM is not completely possible, resolving the components of this mixture requires the use of the substance-specific, molecular-level order mass spectra residing in the (sub)millimass space (mDa), which cannot be accessed by low-resolution mass spectrometers [16]. However, advances in analytical mass spectrometry techniques and in particular the introduction of high-resolution mass spectrometry (HR-MS) in the last 20 years have allowed for high-precision formula assignment of diverse organic compounds based on ultra-high mass accuracy and have led to more sensitive, selective, robust, and repeatable analyses [16–18]. Thus, FT-ICR MS has evolved during the past two decades into a powerful tool to study the molecular composition of small-molecule organic complex mixtures (e.g., DOM in ocean waters and soil organic matter (SOM)) in diverse ecosystems [18].

One analytical approach using FT-ICR MS is direct injection mass spectrometry (DI-MS), which involves the introduction of liquid samples directly into the mass spectrometer without an attached fractionation step. This technique considerably reduces the analysis time, as it is amenable to the use of auto sample handlers, allowing to process hundreds of samples per day. Even though DI-MS has ample coverage and can detect a wide range of compounds (e.g., lipids, sugars, amino acids, or lignin), some drawbacks are its inability to separate chemical isomers, lack of fine resolving power, and most importantly signal suppression or enhancement that can confound downstream data analysis due to ion suppression caused by the injection of all compounds together at the same time [19]. Nonetheless, the use of direct infusion FT-ICR MS (DI FT-ICR MS or just FT-ICR MS) can provide a comprehensive overview of the molecular profile of the NOM and a baseline to understand how biological systems respond to changes in the biotic or abiotic factors acting upon them. A wide range of studies have used FT-ICR MS as a powerful tool to characterize NOM changes in environmental samples [20–23] and its use continues to increase every day.

Numerous tools for signal processing and assignment of molecular formulas of raw FT-ICR MS data exist. They include proprietary tools but also open-source software such as Formularity [24] and most recently CoreMS [25], which provides a comprehensive software framework, including signal processing and sample-agnostic molecular formula assignment. Signal processing and molecular formula assignment steps will ultimately produce large data matrices containing the elemental composition and measured abundance of the peaks present in each sample. These large data sets often require specialized data analysis pipelines for filtering, interrogation, comparison, and visualization [26]. Open-source software and pipelines available for the analysis and visualization of FT-ICR MS data include web-based applications such as UltraMassExplorer (UME) [27], FREDA [28], MetaboAnalyst [29], and DropMS [30]. Even though the graphic user interface (GUI) of these software packages is user-friendly, these types of software can be very restrictive in their use and do not allow users to fully customize their analysis to their research needs. Other more programming-friendly software packages include the visualization tools *i-*van Krevelen [31] and OpenVanKrevelen [32] and the more comprehensive data analysis tools PyKrev [33] and *ftmsRanalysis* [26]. These approaches however require users to be competent in coding and programming in either R (for *ftmsRanalysis*) or Python, which further limits their usefulness for researchers without those skills. Thus, despite the broad availability of software packages for the analysis of FT-ICR MS data, they often incur in a compromise between flexibility/customizability and user-friendliness that we aim to address with MetaboDirect by making it a fully automated pipeline capable of easily generating all the figures, plots, and analysis that are commonly used by the scientific community to visualize, analyze, and interpret FT-ICR MS data sets (Table 1).

**Table 1:** Comparison of the features of MetaboDirect with other available software for the analysis of FT-ICR MS-based metabolomics data sets

	**Feature**	**MetaboDirect**	**MetaboAnalyst**	**PyKrev**	**ftmsRanalysis**	**UME**
**Software appearance**	**User interface**	**Command line**	**Web GUI**	**Python module**	**R package**^**a**^	**Web GUI**
	Platform	Cross platform				
	Language	Python, R	R, Java	Python	R	R
	Coding knowledge required	Minimal^b^	No	Yes	Yes	No
	Open source	✔				
	Documentation	✔				
**Data pre-processing**	Raw spectra processing	✖	✔^c^	✖	✖	✖
	Mol. formula assignment	✖	✔	✖	✖	✔
	Filtering and normalization	✔				
	Thermodynamic indices calculation	✔ (DBE, GFE, AI, NOSC)	✖	✔ (DBE, AI, NOSC)	✔ (DBE, GFE, AI, NOSC)	✔ (DBE)
	Molecular class assignment	✔	✖	✔	✔	No
**Data Exploration**	Van Krevelen diagrams	✔	✖	✔	✔	✔
	Molecular composition plots	✔	✖	✔	✖	✖
	Thermodynamic indices plots	✔	✖	✔	✔	✔
	Chemodiversity indices	✔	✖	✔	✖	✖
	Pairwise comparisons	✔	✖	✖	✔	✖
	Database mapping	✔	✔	✖	✔	✖
**Statistical analysis**	PERMANOVA	✔	✖	✖	✖	✖
	NMDS	✔	✖	✖	✖	✔
	PCA	✔	✔	✔	✖	✖
**Molecular transformation network analysis**	Transformation calculation	✔	✖	✖	✖	✖
	Network construction	✔	✖^d^	✖	✖	✖
	Network Analysis	✔	✖^d^	✖	✖	✖
**Extras**	Customizable plots	✔	Limited	✔	✔	✖
	Multiple grouping variables	✔ (up to 2)	✖	✖	✔	✖

*Abbreviations*: *DBE* Double-bond equivalent, *NOSC* Nominal oxidation state of carbon, *AI* Aromaticity index, *GFE* Gibbs free energy

^a^Also available as the web-app FREDA[28]

^b^No coding experience is required to run MetaboDirect, but some experience in R is needed if fully customized plots are desired

^c^For LC-MS spectra

^d^MetaboAnalyst can construct and analyze networks, but the list of nodes and edges must be provided by the user and are not generated directly by their pipeline

Here, we introduce MetaboDirect, an easy-to-use, command-line-based pipeline for the analysis of direct injection FT-ICR MS-based metabolomic data collected from diverse ecosystems (soil, river, plants, bacterial cultures, etc.), which combines the easy usage of the web-based applications with the flexibility and customizability of the more programming-friendly software currently available. MetaboDirect was designed to facilitate data exploration, data visualization, chemodiversity, and statistical analysis of metabolomic profiles, as well as the generation of transformation networks based on mass differences. The pipeline accepts peak abundance and assigned molecular formula data produced after an initial processing of raw FT-ICR MS spectra or any other high-resolution MS technique. MetaboDirect is designed to run using a single line of code to automatically produce all the analyses, figures, and tables described in its documentation without requiring long computing times (0.5–2 min for the main MetaboDirect pipeline, but longer if transformation networks are calculated). To further ease the access of MetaboDirect to scientists of all programming-skill levels, detailed information regarding its functioning, and available options for data filtering and normalization methods is available through its User’s Guide (https://metabodirect.readthedocs.io/en/latest/).

In this manuscript, we showcase the use and outputs of MetaboDirect through the analysis of two FT-ICR MSdata sets. The first metabolomics data set was generated from an established, ecologically relevant marine phage-host model system [34–36] designed to study new virus-host-nutrient interactions and their impact on the composition of bacterial metabolites [37]. The second metabolomics data set was obtained from a study that aimed at elucidating plant leachate, in particular *Sphagnum fallax* leachate, degradation pathways and biochemical transformation in the presence and absence of microorganisms [12].

### Implementation

#### The MetaboDirect pipeline

The MetaboDirect pipeline was developed in Python 3.8 [38] and R 4.0.2 [39] and is available to install through the Python Package Index (https://pypi.org/project/metabodirect/). It requires the Python dependencies NumPy [40], pandas [41, 42], seaborn [43], py4cytoscape, and matplotlib [44]. This software is compatible and has been tested to function on Windows, Linux, and MacOS. The full documentation for the pipeline can be found on its ReadTheDocs webpage: https://metabodirect.readthedocs.io.

**Fig. 1:**
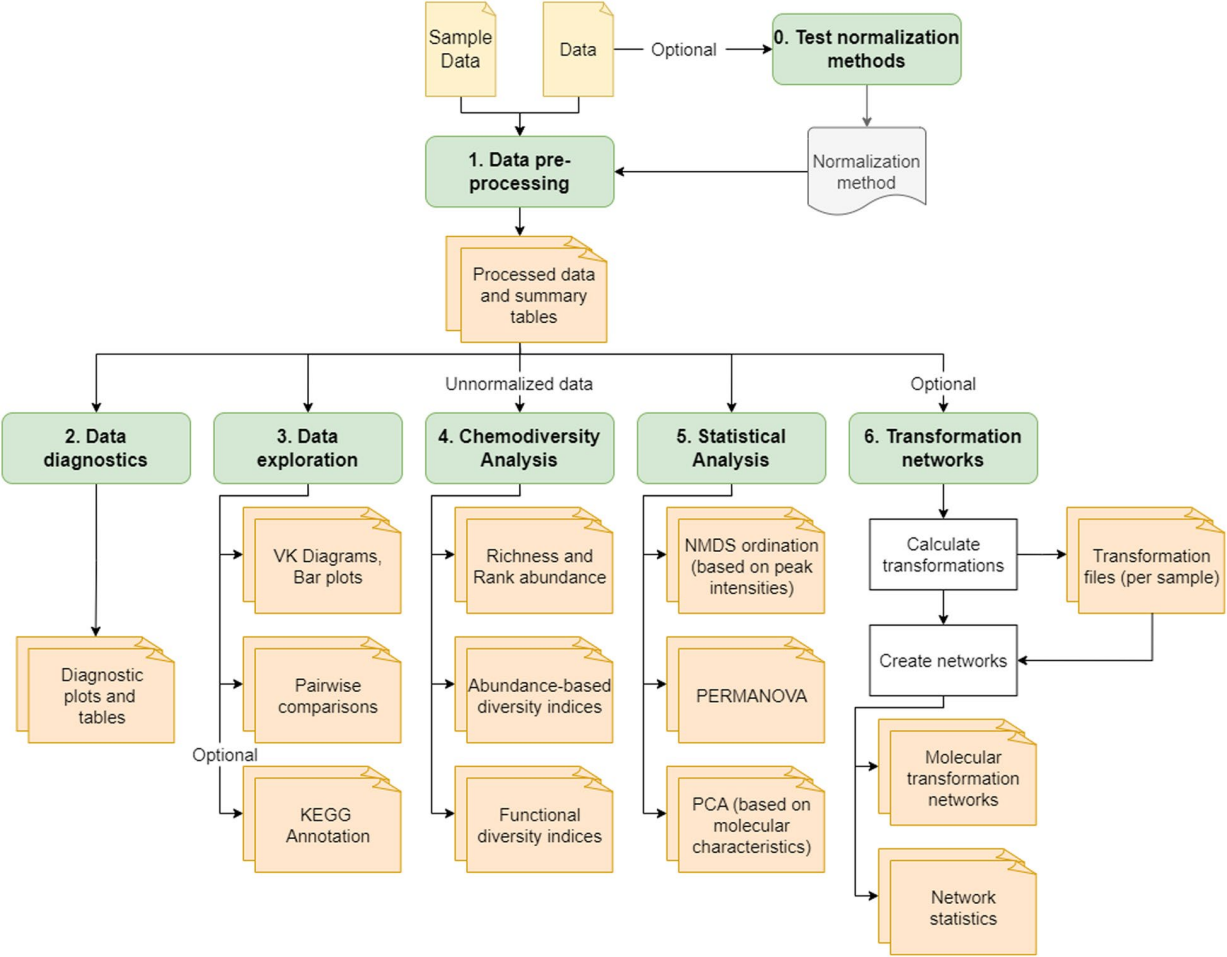
Workflow steps in the MetaboDirect pipeline. The MetaboDirect pipeline consists of six main steps for the analysis of FT-ICR MS data. At each step, several tables and plots will be generated automatically based on the users’ input

The MetaboDirect pipeline consists of 6 major steps/categories (Fig. 1): (i) data pre-processing, (ii) data diagnostics, (iii) data exploration, (iv) chemodiversity analysis, (v) statistical analysis, and (vi) transformation network analysis. All these steps can be run directly with the “metabodirect” command. An additional script, “test_normalization” is included to help users select the best normalization method and can be run before the main MetaboDirect pipeline. Even though MetaboDirect does not provide raw spectra data preprocessing, it has been designed to work with the output file (in *.csv* format) generated directly by Formularity [24] which uses FT-ICR MS data in .xml format. The Formularity output file includes a list of assigned molecular formulas, and their corresponding peak intensities (monoisotopic peaks) and *m/z* values. Because the Formularity algorithm filters formulas based on the “Seven Golden Rules” [45], some peaks will not be assigned a molecular formula. MetaboDirect can work with any list of masses and their corresponding molecular formulas generated by any other software such as DataAnalysis (Bruker Daltonics, Bremen, Germany) or Xcalibur (Thermo Scientific), the open-source CoreMS or a combination of software such as MassSpecWavelet [46] followed by *MFAssignR* [47] as long as the .csv file is formatted according to the MetaboDirect documentation. The selection of the best tool for raw spectra processing will depend on each researcher and their particular dataset [48].

##### Selection of the best normalization method

The companion script “test_normalization” uses the Statistical Procedure for the Analysis of Normalization Strategies (SPANS) [49] to aid in the selection of an appropriate normalization method for the intensities of the detected peaks. This approach has been previously demonstrated to work well with FT-ICR MS data [50] and uses a modified “spans_procedure” function from the R package *pmartR* [51], which evaluates the amount of bias that normalization methods available in MetaboDirect may introduce to the data. The result of this step will be dependent on the nature of the data set and the grouping variable that was analyzed. The calculated SPANS scores for the combination of normalization and subset methods are presented in a heatmap.

##### Data pre-processing

This is the first step of the main MetaboDirect pipeline. During this step, detected peaks are filtered by their *m/z* values (based on the user’s input), isotopic presence (^13^C peaks), error in formula assignment (0.5 ppm), and based on the number of samples that they are present in (threshold determined by the user). Compound classes of each of the filtered peaks are then determined based on the assigned molecular formula using the criteria specified in Supplementary Table 1 [23, 52].

The molecular properties and hypothetical decomposability of the filtered peaks that received a molecular formula assignment are determined by calculating several thermodynamic and molecular indices based on each peak’s elemental composition (equations used to calculate all thermodynamic indices are included in Supplementary Table 2). These indices include nominal oxidation state of carbon (NOSC) that describes the average carbon oxidation state of the assigned peak based on its elemental composition [53], Gibbs free energy (ΔG°C-ox or GFE) that indicates how likely the compound is to be degraded [53], modified Aromaticity Index (AImod) that reflects the “density” of carbon-to-carbon double bonds within a molecule [54, 55], and finally double bond equivalent (DBE) that represents the amount of unsaturation in a molecule and can indicate the presence of aromatic structures [55].

Furthermore, peak intensities are normalized in this step based on the user’s input. Normalization methods available in MetaboDirect are based on the methods used in other similar tools [33, 50], are detailed in Supplementary Table 3, and include “max,” “minmax,” “mean,” “median,” “total sum,” and “zscore.” Even though other normalization methods such as the Probabilistic Quotient Normalization (PQN), Quantile Normalization, and Variance Stabilization have also been used for the analysis of metabolomics data [56], the ones available in MetaboDirect were selected because of their broad use, relative simplicity, and easy-implementation, compared with others such as PQN that requires a conscious selection of a reference spectrum [57], which are not often available for complex samples or present in exploratory analysis.

This pre-processing step generates several .csv files containing the list of filtered peaks with their respective thermodynamic and molecular indices and the normalized and unnormalized intensities which will be used in the next steps of the pipeline.

##### Data diagnostics

This part of the pipeline generates plots of the total number of peaks detected in each sample after filtering (based on previous step) and the number of peaks that received a molecular formula assignment in each sample out of the total number of peaks. Both the total number of peaks and the total number of molecular formulas assigned per sample are reported in .csv tables and plotted as bar plots. Additionally, the data diagnostics step plots the error distribution of assigned molecular formulas as faceted scatterplots.

##### Data exploration

This step produces several plots based on the molecular and thermodynamic properties of the peaks that received molecular formula assignment. During data exploration, MetaboDirect generates van Krevelen diagrams [58] of the peaks with molecular formulas; density and violin plots of the thermodynamic indices calculated in the pre-processing step, including whether or not there is a significant difference between the different groups using Tukey post hoc tests; bar plots with the molecular and elemental composition for each group/treatment; and finally pairwise comparison plots based on the user’s selected grouping variable/s. Pairwise comparisons are used to show which peaks are unique and which are shared between the different groups using van Krevelen diagrams, Venn diagrams, and upset plots. As an additional option available in this step, MetaboDirect can use the assigned molecular formulas to query the KEGG database [59] using the R package *KEGGREST* [60] to provide putative KEGG Pathway, Module, and Brite annotations as .csv files.

##### Chemodiversity analysis

In this step, the MetaboDirect pipeline calculates indices that were originally designed for biological species but can be adapted to other organizational levels [61]. These indices are then applied at a macromolecular diversity level [62]. To this end, raw peak intensities are sum-normalized [13] and used to calculate diversity metrics using functions from the R packages *vegan* [63] and *SYNCSA* [64]. Metrics generated include richness and rank abundance that represent the number of detected metabolites, as well as diversity indices that take into account the evenness of the species abundances (how close are the abundances of each metabolite among samples) or their functional traits [61]. Since the majority of FT-ICR MS spectra are collected using electrospray ionization (ESI) [65], a “soft” ionization technique, with little to no fragment ions observed [66], this approach, while dependent on the total number of peaks detected, is valid especially if all spectra within a given data set are collected using the same instrument parameters. Abundance-based diversity is measured with the Shannon diversity index [67], the Gini-Simpson index [68, 69], and the Chao1 richness estimator [70]. Functional-based diversity, based on the compound’s elemental composition, potential decomposability, and unsaturation/aromaticity, is measured with Rao’s quadratic entropy index [71]. All diversity indices are visualized as box plots grouped by the user’s defined grouping variables and exported as .csv files.

##### Statistical analysis

In the data statistical analysis step, the normalized intensities of the peaks (from pre-processing step) are transformed into Bray-Curtis, Euclidean, or Jaccard distances (depending on the selected normalization method) using the “vegdist” function for the *vegan* package and then used to perform a permutational analysis of variance (PERMANOVA) [72]. The ordination of the data, based on the normalized intensities, is calculated using non-metric multidimensional scaling (NMDS) [73], as it provides a robust approach and can use any of the dissimilarity (distances) metrics mentioned before [74]. NMDS scores are then visualized as scatter plots using the first two components as axis, while the results of the PERMANOVA are exported as a .csv file. Additional ordination of the data is provided as Principal Component Analysis (PCA) [75] scree plots and biplots based on the molecular composition and magnitude-averaged thermodynamic indices of the samples [76].

##### Transformation networks

This is an optional step of the MetaboDirect pipeline as it is time-consuming and, in most cases, is only needed to be run once per each data set. Molecular transformation networks for each sample (mass difference network-based approach) are generated in this step, where nodes represent peaks detected in the different samples and edges represent the putative chemical transformations happening between the nodes [21, 77, 78]. This step consists of two main processes: the calculation of mass-based chemical transformations and the creation of the molecular transformation networks.

Because identification and annotation of specific compounds cannot be done with FT-ICR MS, this analysis utilizes molecular mass-based transformations that are determined by calculating the differences between the *m/z* of all peaks present in each sample and comparing them to the list of pre-defined masses of common metabolic reactions (biochemical transformations key) [77]. Mass differences with a maximum error of 1 ppm against the reference biochemical transformation keys are kept as a putative mass-based transformation, as such small differences are unlikely to be observed by chance, and thus, they may have chemical significance [77]. The transformations included in the predefined biochemical transformation key are further classified in biotic or abiotic transformations based on a previous study in peat bogs [12]. If preferred, the authors recommend users to create a user-specific biochemical transformation list that can be used in this step. This user-specific biochemical transformation may contain commonly observed biochemical transformations for the analyzed system or those that the users are interested in. The results are exported as .csv “edge” files containing the potential transformations occurring between the masses in each sample. Additional files with the number of transformations occurring per sample are also generated. Networks are then constructed using Cytoscape [79] and colored based on their molecular class. Transformation networks can then be used to inform the chemical connections between the detected compounds. Furthermore, network statistics will be calculated and reported as .csv tables and bar plots.

#### Pipeline testing

The performance of the MetaboDirect pipeline was tested here using two previously analyzed FT-ICR MS data sets collected in negative ion mode. The first came from the exometabolome of a marine phage-host model system that uses a known, ecologically relevant marine bacterium (*Pseudoalateromonas*) and two contrastingly different infecting phages (podovirus HP1 and siphovirus HS2) that have been extensively characterized via genomics and time-resolved transcriptomics and proteomics under nutrient-rich conditions [34–37]. Since viruses control microbes that provide essential ecosystem services through infection and reprogramming of the host cell metabolism, they can impact the composition of bacterial exometabolites in the ecosystem. This data set is used here to show how MetaboDirect can help develop foundational approaches to studying how viral infection of bacterial communities could impact ecosystem outputs with significant repercussions on ecosystem functioning. Specifically, MetaboDirect was applied to determine how different infecting phages influence the metabolites produced under nutrient (P) rich conditions [37].

The second data set was obtained from an incubation experiment of *S. fallax* leachate that was conducted in the presence and absence of microorganisms [21]. This data set was used to demonstrate how MetaboDirect can be used to help elucidate how organic matter degradation pathways can change in the presence or absence of microbial communities.

Additionally, we benchmarked the compute time required for the main pipeline of MetaboDirect to be completed for mock data sets differing in their number of samples and the average number of metabolites that were assigned a molecular formula. Mock data sets were generated by randomly subsampling unpublished data.

It is important to note that the goal of this paper is to provide two comprehensive examples of the application of the MetaboDirect pipeline, contrasts its capabilities against other available software, and benchmarks its “clock times” (i.e., compute time), but not necessarily to provide biological interpretations of the data analyzed. Information regarding the incubation parameters, soil organic matter extraction protocols, and high-resolution mass spectrometry data collection is provided in the Supplementary information.

#### Code availability

The complete code for the MetaboDirect pipeline is freely available at its GitHub repository: https://github.com/Coayala/MetaboDirect.

## Results and discussion

Here, we demonstrate MetaboDirect’s functionality in data processing, filtering, and visualization (Fig. 1) through the analysis of two distinct FT-ICR MS data sets. The bacterium-phage model system data set included metabolites or mass spectrometry peaks present in 36 samples from bacteria infected by two different phages and a control treatment under nutrient-rich conditions at different time points. The *S. fallax* leachate incubation metabolomics data set consisted of 4 samples, two samples where the plant leachate was incubated anaerobically in the presence of in situ microbial communities and two samples where the plant leachate was incubated in the absence of any microbial communities.

### MetaboDirect pipeline testing

One of the advantages of the MetaboDirect pipeline is that it allows to automatically run all the analysis at once in a reproducible manner. The main steps of the MetaboDirect pipeline (steps I through V) run quickly, facilitating the rapid exploration of the data using different user-defined parameters in an efficient manner. When the compute time of MetaboDirect was benchmarked without its optional steps (KEGG mapping and the construction of transformation networks using data sets of different size), 40 samples were processed in less than 1 min whereas 120 samples took as little as 2 min to generate all the figures, plots, and outputs discussed above at once. Even though the variation in execution time in MetaboDirect depends on both the number of samples analyzed, and the number of molecular formulas assigned per each sample (Supplementary Fig. 1), the MetaboDirect pipeline is still superior to other FT-ICR MSsoftware where the user is expected to plot the data one sample at a time and one figure type at a time.

For the bacterium-phage data set a “report” file produced by Formularity [24] was processed through the whole MetaboDirect pipeline. The data set had an average of 1025 peaks detected across the whole data set (*n* = 36 samples) and an average of 495 peaks that got assigned a molecular formula. The main steps of the MetaboDirect pipeline (without KEGG database mapping or calculating transformation networks) took less than 1 min (~36 s) for this data set. The analysis including KEGG mapping and calculation of the putative biochemical transformations took around 10 min. The full analysis includes KEGG mapping, calculating the putative biochemical transformations and creating the networks took about 21 min. For the *S. fallax* data set, the data was obtained from the paper supplementary tables [12] and rearranged to fit the format needed for MetaboDirect. This was a smaller data set (*n* = 4 samples) with an average of 1793 assigned molecular formulas across the whole data set. For this data set, the main steps of the MetaboDirect pipeline were clocked at around 30 s. However, due to the larger number of assigned molecular formulas, a full analysis of this data set (including KEGG mapping and construction of molecular networks) took about 32 min.

Outside MetaboDirect, KEGG mapping is usually performed using KEGG MAPPER [80], a tool available directly through the KEGG website. While this tool searches various KEGG objects, including genes, KOs, EC numbers, metabolites, and drugs, against KEGG pathway maps, it requires the user to first identify the KEGG ID of each metabolite before manually importing the data to this MAPPER tool. MetaboDirect on the other hand performs all these steps automatically. The generation of biochemical transformations is currently done using the Cytoscape app MetaNetter_2 [81] that can only generate one network at a time, and the user has to provide three files for each sample at a given time and manually set up network generation parameters such as mass tolerance. These files include (1) a list of accurate masses per sample from the FT-ICR MS data, (2) a list of accurate masses of common biochemical transformations that the user is interested in quantifying within the sample, and (3) a metadata file that includes the different characteristics of each accurate mass. MetaboDirect on the other hand performs all these analyses at once and for all samples within a given data set.

For both data sets, the first step was to use the “test_normalization” companion script to help determine which normalization method worked the best. FT-ICR MS data typically carries high biological or technical variation, and normalization is the first required step to enable data quantification. Proteomic analyses have shown [82] that normalization methods are data set-dependent. As such, MetaboDirect relies on the use of SPANS to identify an appropriate normalization method for each data set that can improve the structure of the data without introducing bias [49]. It is important to note that even though SPANS have been shown to be an appropriate strategy for selecting normalization methods for FT-ICR MS data sets [50], it may not be appropriate for data sets with systematic differences in intensity distributions among the groups of interest [49, 50]. In this case, the user may test various normalization strategies and choose the one that works the best.

Median and *z* score normalization appeared to be the appropriate normalization methods for the test data sets as they had the highest SPANS scores respectively (Supplementary Fig. 2). Thus, *z score* normalization was selected for the bacterium-phage data set and *median* for the *S. fallax* data set for data normalization and statistical analysis in the subsequent steps.

Following normalization testing, the data pre-processing step was used to filter out specific peaks and to identify any problems within the data set. In the bacterium-phage data set, ~200 masses were filtered out from the data set because they were assigned molecular formulas containing an isotopic carbon. The diagnostics step further identified one sample with a very low number of assigned molecular formulas compared to the other samples, which can be a potential outlier for the analysis (Supplementary Fig. 3). No peak was filtered in the *S. fallax* data set as it was previously filtered by the authors of that study.

Following the data pre-processing, the data exploration step was used to provide an overview of the molecular composition and thermodynamic characteristics of each of the user-defined groups for each data set: the type of bacteriophage (HP1 vs. HS2 vs control) or the treatment (control vs inoculation). The use of the molecular properties and thermodynamic indices calculated by MetaboDirect are useful in studying how biotic and abiotic factors can influence the metabolites’ lability (NOSC and ΔG°C-ox) and degree of saturation of the metabolites present in each set of samples [20, 83].

Exploratory analysis of the bacterium-phage data set showed that the chemical composition of the exometabolome of cells, infected with the HS2 phage, changed at 30 min post-inoculation, but then, it returned to be similar to the chemical composition of the uninfected cells (Supplementary Fig. 4A), while the molecular composition of exometabolome from cells infected with the HP1 phage did not change throughout the experiment. This change in molecular composition resulted in changes in the modified aromaticity index (AI_mod), and the double-bond equivalent (DBE) remains similar to the other treatments, showing a possible thermodynamic redundancy between the infected and uninfected cells.

**Fig. 2:**
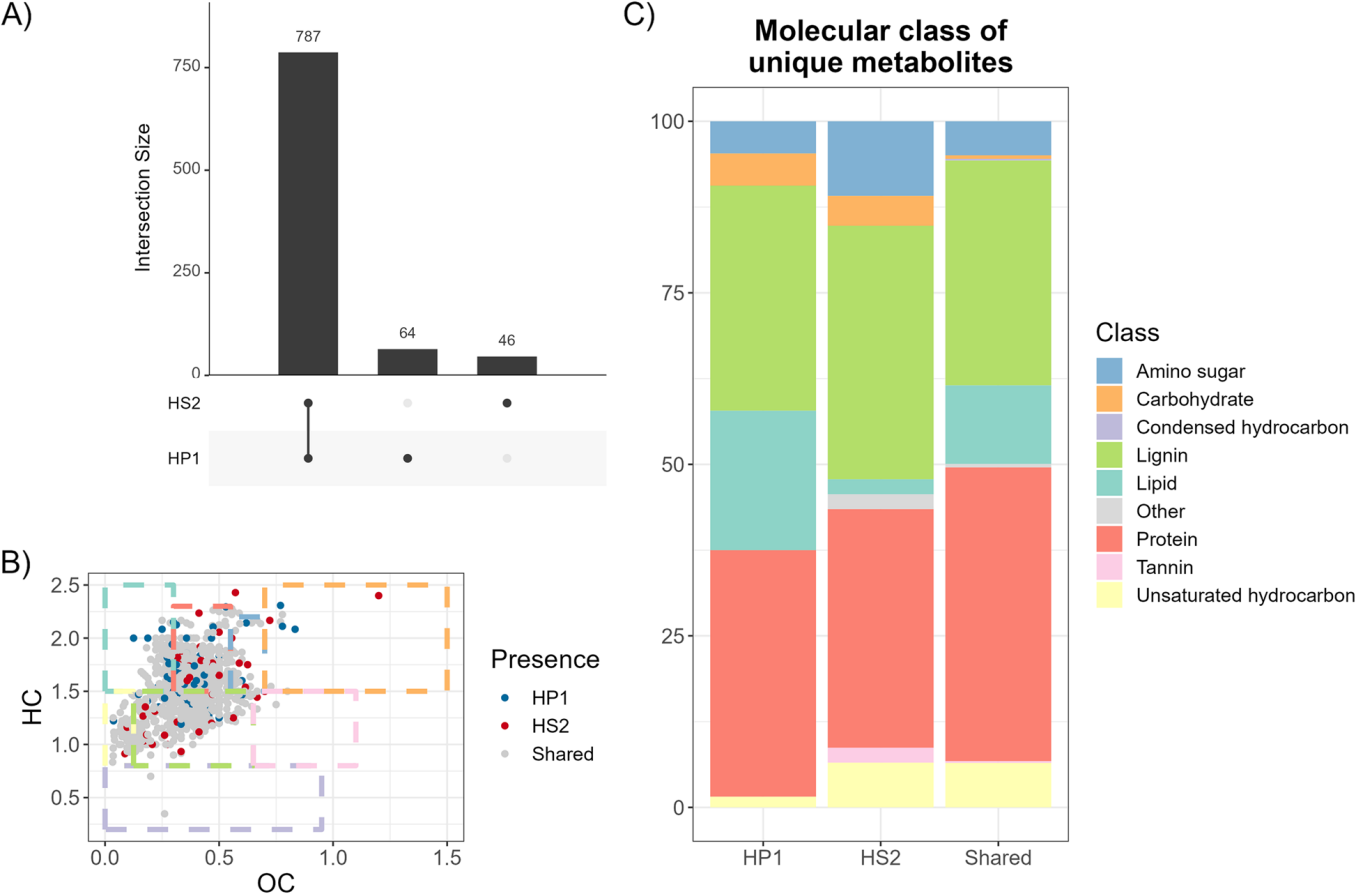
Exploratory plots of the bacterium-phage data set.** A** Upset plot showing the number of metabolites that are shared and unique between the uninfected and infected cells. **B** Van Krevelen diagram showing metabolites that are shared and unique between cells infected with the two different phages, HP1 and HS2. **C** Molecular composition of the unique metabolites showing that there are unique protein-like, lignin-like, and lipid-like metabolites in cells infected with each phage

MetaboDirect automatically generates all pairwise comparisons for the selected grouping factors being analyzed. In this manner, the pipeline allows to identify which metabolites are common between the different conditions and which metabolites are unique to each treatment. For the bacterium-phage data set, this analysis showed that most detected metabolites were shared between the infected and uninfected cells (Fig. 2A) and that the unique metabolites were mostly protein-like, lignin-like, and lipid-like metabolites (Fig. 2B, C). Conversely, pairwise comparisons of the *S. fallax* data set showed that the number of unique metabolites in the control and the inoculated samples was almost the same as the number of shared metabolites (Supplementary Fig. 5A), with lignin-like metabolites being most of the unique metabolites in the inoculated samples and tannins being present only on the control samples (Supplementary Fig. 5B).

Diversity metrics, originally designed to study ecological species, have been adapted to analyze other systems, such as metabolites at the macromolecular level [62]. In this case, molecular formulas are akin to individual species, while their peak intensity is used for abundance [84]. Commonly used diversity metrics in the study of metabolite assemblages include the measure of molecular richness (based only on the number of molecular formulas) [13, 14, 85]; the use of abundance-based diversity metrics such as Shannon, Gini-Simpson [84, 86, 87], or the Chao 1 [13, 14] indices, which (based on the richness and the relative intensity of the metabolites); as well as the use Rao’s quadratic entropy to measure functional-based diversity [84, 87] (which uses different molecular characteristics as traits). MetaboDirect assists with calculating and visualizing all the diversity indices mentioned above.

Chemodiversity analysis for the bacterium-phage data set found little differences in the metabolite diversity between the infected and uninfected cells for either abundance-based diversity or functional diversity (Supplementary Fig. 6A, C). For the *S. fallax* incubation data set, inoculating the *S. fallax* leachate with microorganisms increased the diversity of the metabolites (i.e., richness) (Supplementary Fig. 6B) but decreased the functional diversity, suggesting that the metabolites in the inoculated samples were less diverse in terms of their decomposability or reactivity, aromaticity, and elemental composition (Supplementary Fig. 6D).

Multivariate analysis such as NMDS, PCA, and PERMANOVA can be used to better understand the relationships of the normalized peak intensities or the molecular characteristics of the metabolites and the biotic and abiotic factors, as well as to find trends among a large number of samples at the same time [13, 68, 88]. We used these methods to try to illustrate the effect of phage type and the time of the incubation on the relative amount and molecular characteristics of the metabolites produced by the bacterial-phage system during infection. Even so, the type of phage (or lack thereof) infecting the cells seems to produce a significant difference in the metabolite/organic compound content of the samples (PERMANOVA *p* value < 0.05, Supplementary Fig. 7C). The ordination analysis was not able to discriminate among the sample groups suggesting that the differences are subtle (Supplementary Fig. 7A and B).

**Fig. 3:**
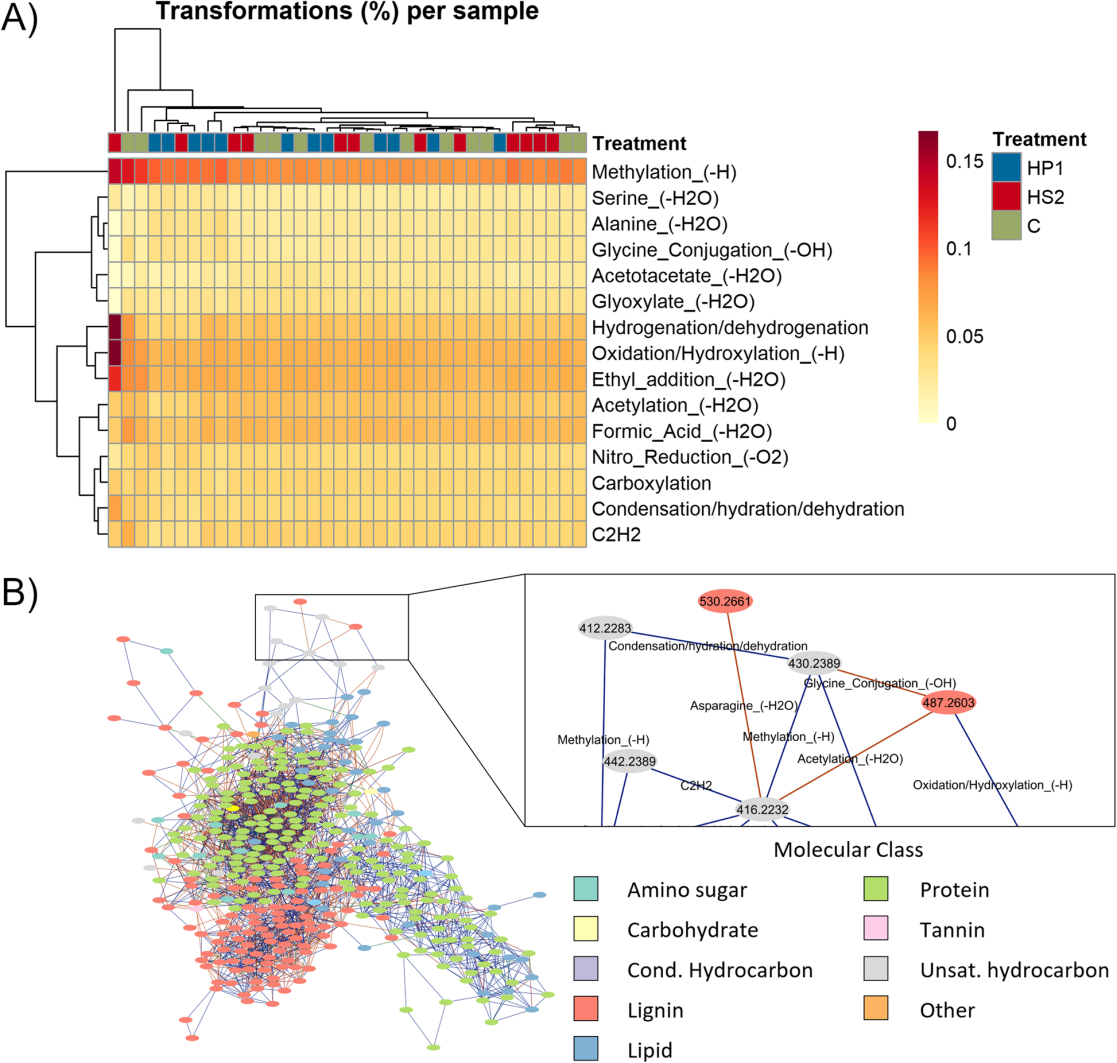
Mass-based transformation network analysis of the bacterium-phage data set.** A** Heatmap with the top 15 more abundant transformations among all the samples converted into a percentage of the total number of transformations in each sample. **B** Molecular network for the sample P_rich_C_T30_R3. Like most of the other samples, the transformation network showed a cluster of lignin-like metabolites interacting with a cluster of protein-like metabolites. The zoomed panel at the left shows that the transformation networks have the masses of each sample as nodes, while the edges between those nodes represent the transformations

The last step of MetaboDirect produces molecular transformation networks for each of the samples. These networks are generated ab initio from the masses that are determined through high-resolution mass spectrometry and are based on the fact that the ultra-high mass accuracy of the method allows for identifying chemically transformed species using clearly defined mass differences [77, 78]. The number of transformations can be used to quantify the differences in the microbial metabolic pathways between different groups and to identify potential hub metabolites that are involved in many pathways and reactions and that can be important for the regulation of the studied system. The type of biochemical transformations on the other hand provides information on the type of reactions that are occurring within a given sample/system that take place either through enzymes whose presence can be validated through other omics analysis or through non-enzymatic metabolite interconversions within the cell/system that we usually do not account for in most microbiome studies. For the bacteriophage data set, the most abundant chemical transformation was methylation (i.e., loss or gain of a methyl group). However, we did not observe significant changes in the quality and quantity of all biochemical transformations with infection. Interestingly, analysis of the transformation networks produced by MetaboDirect for the bacterium-phage data set showed that for most of the samples, there was always a cluster of protein-like metabolites interacting with phenol (lignin)-like metabolites (Fig. 3B) suggesting that these ab initio networks will be very useful for future scientific discoveries especially when such studies are complemented with other omics data sets.

### Comparison with other available software

MetaboDirect was designed for reproducible analysis of direct injection FT-ICR MS data, ranging from diagnostics and data exploration to chemodiversity and statistical analysis (Table 1), and it is fully automated with a single line of code and offers an automated pipeline for the generation and visualization of mass-based transformation networks. Previously, this type of analysis was mostly performed through the use of the Cytoscape plugin MetaNetter_2 [81], in a laborious and time-consuming process which involves exhaustive file preparation as it must be done on a per sample basis. While some software packages with a GUI, such as the web-based apps MetaboAnalyst, UltraMassExplorer, and FREDA are user-friendly, they are restricted to some of the most common analysis tools for FT-ICR MSdata such as the generation of van Krevelen plots and multivariate statistical analysis, and they lack customization. On the other hand, more comprehensive software packages, which are presented in the form of libraries for commonly used programming languages, such as *PyKrev* and *ftmsRanalysis*, allow the users to deeply customize their data analysis. However, they require medium to advanced skills in computer programming to be able to take full advantage of their parameterization.

MetaboDirect stands in the middle of those groups, requiring minimal coding experience, as all the output files are obtained using a single line of code. Moreover, MetaboDirect provides the user with all the R scripts used in the generation of all the tables and visualizations, thus allowing the user to fully customize any of the figures and tables produced by MetaboDirect and to use that data in any additional analysis. Therefore, MetaboDirect can be attractive to users with all programming skill levels, allowing them to take advantage of a fully automated pipeline that can be easily customizable if needed.

As observed in Table 1, MetaboDirect can perform all the analyses offered by the other available software for FT-ICR MS data, except for “raw spectra processing” and “molecular formula assignment”. Nonetheless, these first two steps in the processing of FT-ICR MS data can be easily achieved by using Formularity, CoreMS, the R packages MassSpecWavelet and *MFAssignR*, or vendor software such as the SmartFormula Calculator (Bruker) or the molecular formula assignment module within Xcalibur (Thermo Fisher).

## Conclusion

The use of high-resolution mass spectrometry, specifically FT-ICR MS, to characterize the molecular composition of NOM in different systems is increasing quickly, and thus, the development of reproducible open-source tools for the analysis of such data is urgently needed. Here, we present MetaboDirect, a user-friendly, accessible, and highly comprehensive tool for scientists working to characterize how different biotic and abiotic factors influence organic compound composition in diverse settings and systems that can be used to provide a quick overview of the data, upon which more in-depth analysis can be built. The highly reproducible nature of the analysis provided by MetaboDirect, coupled with the detailed user manual, will allow scientists of all skill levels to fully explore and work with FT-ICR MS data. This in return will greatly facilitate the integration of metabolomics within current microbiome studies and advance our knowledge of how microbial communities influence and are influenced by the chemical makeup of the surrounding system.

## Supplementary Information

**Additional file 1.** Materials and methods used for extracting metabolites and acquiring mass spectrometry data for both data sets.

**Additional file 2: Table S1.** O/C and H/C ratios used to assign putative molecular classes to the detected metabolites. **Table S2.** Equations used to calculate thermodynamic and molecular indexes based on the assigned molecular of the detected mass spectrometry peaks based on their m/z values. **Table S3.** Normalization methods available in MetaboDirect.

**Additional file 3: Fig. S1.** Compute times of MetaboDirect for data sets with different numbers of samples and different numbers of peaks assigned a molecular formula for any given data set. **Fig. S2.** SPANS score calculated by the “test_normalization” companion script for A) the bacterium-phage data set and B) the Sphagnum fallax data set. The x-axis shows the available normalization methods within MetaboDirect, while the y-axis shows multiple combinations of the subset methods with different subset parameters. The SPANS score is shown as a color scale with yellow as the highest score. For more information consult the User’s Guide (https://metabodirect.readthedocs.io). **Fig. S3.** Number of detected peaks that were assigned a molecular formula in the bacterium-phage data set. The sample P_rich_HS2_T30_R4 has few peaks that were assigned a molecular formula and can be a potential outlier during the study. **Fig. S4.** A) Changes in the molecular class composition of the bacterium-phage exometabolome during the incubation. A reduction in the percentage of lignin-like compounds is observed at 30 minutes after inoculation only for the HS2 phage. B) Violin plot of the changes in the aromatic index reflects the changes in molecular composition, cells infected with the HS2 phage have lower AImod at 30 minutes after inoculation (Tukey HSD test, p-value < 0.05). C) Violin plot showing that double bond equivalence (DBE) of HS2 is reduced after 30 minutes and remains low until the end of the experiment (Tukey HSD test, p-value < 0.05). For B), C) and D) * (p-value < 0.05), ** (p-value < 0.01), *** (p-value < 0.001), **** (p-value < 0.0001). **Fig. S5.** A) Upset plot showing the number of metabolites that are shared and unique between control and inoculated treatments of the S. fallax leachate. B) Van Krevelen diagram showing metabolites that are shared and unique between control and inoculated treatments of the S. fallax leachate. C) Molecular composition of the unique metabolites showing that there are unique protein-like, carbohydrate-like, lignin-like and lipid-like metabolites. **Fig. S6.** Results of the chemodiversity analysis of the FT-ICR MS data. A) and B) Abundance-based diversity metrics including the Chao1 richness estimator, Gini-Simpson, and Shannon indexes. C) and D) Functional-based diversity using Rao’s quadratic entropy using different traits: Elemental composition is based on the number of elements in each molecular formula. Insaturation and aromaticity uses DBE and AImod as traits. Reactivity uses Gibbs’ free energy as a trait. **Fig. S7.** Multivariate statistical analysis performed by MetaboDirect. A) NMDS plot showed a small clustering of samples based on the content of phosphorus rather than the type of infection, as denoted by the clusters of colored dots B) PCA plots by compound molecular class. Like the NMDS plot, there was no clustering of the sample neither by phage nor time. C) PERMANOVA result, the last column shows the p-value of the analysis. There was not a significant effect of the phage or the time.

## Data Availability

(1) Mass spectrometry data in XML format and peak abundance list with molecular formula assigned for both experiments (formatted to be used in MetaboDirect) are available through the Open Science Framework (OSF) in the following link: https://doi.org/10.17605/OSF.IO/XFHZ9. (2) The original *Sphagnum* data set [12] is available here (https://agupubs.onlinelibrary.wiley.com/action/downloadSupplement?doi=10.1029%2F2020JG006079&file=2020JG006079-sup-0007-Table+SI-S02.xlsx). The code of the MetaboDirect pipeline used for this analysis (v0.3.4) is available in its GitHub repository (https://github.com/Coayala/MetaboDirect) with https://doi.org/10.5281/zenodo.7278253.

## Abbreviations

DOM: Dissolved organic matter
FT-ICR MS: Fourier-transform ion cyclotron resonance mass spectrometry
GUI: Graphical user interface
NMDS: Non-metric multidimensional scaling
NOM: Natural organic matter
PCA: Principal component analysis
SOM: Soil organic matter
