# Supplementary material for: MetaboDirect: an analytical pipeline for the processing of FT-ICR MS-based metabolomic data
Source: Microbiome. 2023 Feb 17;11:28. doi: 10.1186/s40168-023-01476-3 (PMC9936664; doi:10.1186/s40168-023-01476-3)
Supplement: Supplementary file 2 — Additional file 1. Materials and methods used for extracting metabolites and acquiring mass spectrometry data for both data sets. [file 40168_2023_1476_MOESM1_ESM.docx]

# SUPPLEMENTARY METHODS

**Bacterium-phage data set**

The experimental setup from which the data was obtained consisted of a total of three treatments with twelve replicates each (4 replicates x 3 time points). Treatments included bacteria treated with one of two bacteriophages (HP1 or HS2) or with neither of them (control) growing under nutrient (P) rich conditions. Growth and infection were conducted as described previously [37]. Briefly, *Pseudoalteromonas sp* 13-15 were grown shaking at 150 rpm at 21°C in 1%Z+CNP media for P-rich or 1%Z+CN for P-poor. These consisted of 26g sea salts per L, 1% Zobell (26g sea salts, 1g yeast extract, 5g proteose peptone per L), 8.3mM ammonium sulfate, and 0.15mM phosphoric acid (in P-rich medium only), with 11mM glucose added after autoclaving. One colony was inoculated into 10mL and grown overnight before 5x10^8^ cells were transferred to 200mL in 1 L flasks and grown to mid- to late-exponential phase. Then, 1x10^8^ cells were transferred in triplicate to a 1.5mL tube and the volume was adjusted to 1mL. Samples were incubated for 15 min after phage addition at a multiplicity of infection of ~5, and infections were diluted 10-fold in a 1L bottle with fresh medium. From the infections and controls, three samples were collected at 0-, 30- and 60-min post dilution of infection and were 0.2-uM filtered.

**Sample preparation.** Filtered exometabolome aqueous samples were subjected to solid phase extraction (SPE) to remove any salts that can interfere with ionization during mass spectrometry analysis. Briefly, samples were acidified to pH 2 using 1M HCl to enhance extraction efficiency. Acidified samples were then passed through a 3 mL Bond Elut PPE cartridge (Agilent), previously prepped with 3 mL of laboratory grade MeOH, connected to a vacuum. After passing all the samples, cartridges were washed with 3 mL of a 0.01 M HCl solution. The washing step was repeated five times. Washed cartridges were removed from the vacuum and dried using filtered air. Finally, samples were eluted into 2 mL glass vials with 1.5 mL of MeOH and stored at -80 °C until used.

**Direct injection FT-ICR-MS Data Acquisition.** A Bruker 9.4-Tesla, coupled to a standard Bruker electrospray ionization (ESI) source, was used to collect high-resolution mass spectrometry data of the exometabolome filtrates. Before data collection, the instrument was tuned using a Suwannee River fulvic acid (SRFA) standard, purchased from the International Humic Substances Society (IHCC). All samples, standards, and blanks (HPLC grade methanol) were injected directly to the ESI source. A flushing with a mixture of water and methanol was performed in between each sample to prevent carry over. Ion accumulation time (IAT) varied from sample to sample to account for differences in C content. Spectra for each sample was obtained as an average of 144 individual scans. DataAnalysis, version 4.2, (BrukerDaltonik) was used to extract a list of *m/z* values from raw spectra files using the FT-ICR MS peak picker module with a signal-to-noise ratio (S/N) threshold of 7 and the default absolute intensity threshold of 100. Spectra were internally calibrated using an organic matter homologous series separated by 14 Da (CH_2_ groups).

***Sphagnum fallax* data set**

The experiment that collected this data consisted of 12 glass vials filled with filtered *Sphagnum fallax* leachate and flushed with N_2_ to remove microorganisms and maintain anaerobic conditions respectively. Half of the vials were then inoculated with the microbiome obtained from the superficial peat below the point of collection of *S. fallax*. Vials were incubated at 22 °C for two months. A single vial for each treatment (control and inoculation) was sacrificed after one week and then at least every 7-14 days for various measurements including FT-ICR MS.

**Direct injection FT-ICRMS Data Acquisition.** Around 100 uL of DOC obtained from the incubations was mixed with methanol (1:2) before injection to enhance ionization. Samples were injected in a 12 Tesla Bruker FT-ICR MS coupled with a standard Bruker electrospray ionization (ESI) source. Instruments settings were first optimized using the SRFA standard purchased from the IHCC. Blanks consisting of HPLC grade methanol were injected at the beginning and end of each day to monitor potential carry over. The instrument was flushed between samples using a mixture of water and methanol. Ion accumulation time (IAT) varied from sample to sample to account for differences in C content. Spectra for each sample was obtained as an average of 144 individual scans. DataAnalysis, version 4.2, (BrukerDaltonik) was used to extract a list of *m/z* values from raw spectra files using the FT-ICR MS peak picker module with a signal-to-noise ratio (S/N) threshold of 7 and the default absolute intensity threshold of 100. Spectra were internally calibrated using an organic matter homologous series separated by 14 Da (CH_2_ groups).

**Molecular formula assignment**

For both data sets, molecular formula assignment was done using the software package Formularity [12]. Formulas were assigned using the following criteria: S/N > 7, mass measurement error < 1 ppm, C, H, O, N, P and S as the only viable elements, and P required the presence of at least four O. For peaks with large mass ratios (*m/z* > 500 Da), formulas were assigned through propagation of CH_2_, O and H_2_ homologous series. For peaks with multiple molecular formula candidates, the formula with the lowest error and the lowest number of heteroatoms was picked.

**Testing the MetaboDirect pipeline**

**Bacterium-phage data set**

Normalization methods that best represent the differences due to Treatment were tested with the command:

test_normalization Report_fixed.csv metadata_viro.csv Treatment

Then, the main MetaboDirect pipeline was executed with the command:

metabodirect Report_fixed.csv metadata_viro.csv -o md_test_viro -g Treatment -f P_content rich -n zscore -t -k

This command took the Report file from Formularity and filtered only the samples that were grown in P-rich conditions (-f P_content rich), data was normalized with the “zscore” method (-n zscore), molecular formulas were searched against the KEGG database (-k) and transformations were calculated (-t).

Finally, transformation networks were constructed with Cytoscape using the command

create_networks md_test_viro metadata_viro.csv Treatment

***Sphagnum fallax* data set**

Normalization methods that best represent the differences due to Treatment were tested with the command:

test_normalization sphagnum_data.csv metadata_sphagnum.csv Type

Then, the main MetaboDirect pipeline was executed with the command:

metabodirect sphagnum_data.csv metadata_sphagnum.csv -o md_test_sphag -g Type -n median -t -k

This command took a file formatted as mentioned in the User’s Guide. Data was normalized with the “median” method (-n median), molecular formulas were searched against the KEGG database (-k) and transformations were calculated (-t).
