# Supplementary material for: MetaboDirect: an analytical pipeline for the processing of FT-ICR MS-based metabolomic data
Source: Microbiome. 2023 Feb 17;11:28. doi: 10.1186/s40168-023-01476-3 (PMC9936664; doi:10.1186/s40168-023-01476-3)
Supplement: Supplementary file 3 — Additional file 2: Table S1. O/C and H/C ratios used to assign putative molecular classes to the detected metabolites. Table S2. Equations used to calculate thermodynamic and molecular indexes based on the assigned molecular of the detected mass spectrometry peaks based on their m/z values. Table S3. Normalization methods available in MetaboDirect. [file 40168_2023_1476_MOESM2_ESM.docx]

# SUPPLEMENTARY TABLES

## Supplementary Table 1. O/C and H/C ratios used to assign putative molecular classes to the detected metabolites

| **Molecular Class** | **O/C ratio** | | **H/C ratio** | |
| --- | --- | --- | --- | --- |
|  | **Min** | **Max** | **Min** | **Max** |
| Lipid | 0 | 0.3 | 1.5 | 2.5 |
| Unsaturated hydrocarbon | 0 | 0.125 | 0.8 | 2.5 |
| Protein | 0.3 | 0.55 | 1.5 | 2.3 |
| Amino sugar | 0.55 | 0.7 | 1.5 | 2.2 |
| Carbohydrate | 0.7 | 1.5 | 1.5 | 2.5 |
| Lignin | 0.125 | 0.65 | 0.8 | 1.5 |
| Tannin | 0.65 | 1.1 | 0.8 | 1.5 |
| Condensed hydrocarbon | 0 | 0.95 | 0.2 | 0.8 |

## Supplementary Table 2**.** Equations used to calculate thermodynamic and molecular indexes based on the assigned molecular of the detected mass spectrometry peaks based on their *m/z* values

| **Thermodynamic Index** | **Formula** |
| --- | --- |
| Nominal oxidation state of carbon (NOSC) | $NOSC= -\frac{4C+H-3N-2O+5P-2S}{C}+4$ |
| Gibbs free energy (ΔG°C-ox) | $\Delta G^{\circ}C=60.3-28.5*NOSC$ |
| Modified aromaticity index (AImod) | $AImod=1+C-\frac{O}{2}-S-\frac{\frac{H+P+N}{2}}{C-O-S-N-P}$ |
| Double bond equivalence (DBE) | $DBE=1+\frac{2C-H+P+N}{2}$ |

## Supplementary Table 3. Normalization methods available in MetaboDirect

| **Normalization Method** | **Formula** |
| --- | --- |
| max | ${NormIntensity}_{i,j}=\frac{I_{i, j}}{{max(I)}_{i}}$ |
| minmax | ${NormIntensity}_{i,j}=\frac{I_{i, j}-{min(I)}_{i}}{{max(I)}_{i}-{min(I)}_{i}}$ |
| mean | ${NormIntensity}_{i,j}=\frac{I_{i, j}-{mean(I)}_{i}}{{max(I)}_{i}-{min(I)}_{i}}$ |
| median | ${NormIntensity}_{i,j}=\frac{I_{i, j}-{median(I)}_{i}}{{max(I)}_{i}-{min(I)}_{i}}$ |
| sum | ${NormIntensity}_{i,j}=\frac{I_{i, j}}{\sum I_{i}}$ |
| zscore | ${NormIntensity}_{i,j}=\frac{I_{i, j}-{mean(I)}_{i}}{{std.dev(I)}_{i}}$ |
| binary | $presence=1; absence=0$ |
