# Supplementary material for: MetaboDirect: an analytical pipeline for the processing of FT-ICR MS-based metabolomic data
Source: Microbiome. 2023 Feb 17;11:28. doi: 10.1186/s40168-023-01476-3 (PMC9936664; doi:10.1186/s40168-023-01476-3)
Supplement: Supplementary file 4 — Additional file 3: Fig. S1. Compute times of MetaboDirect for data sets with different numbers of samples and different numbers of peaks assigned a molecular formula for any given data set. Fig. S2. SPANS score calculated by the “test_normalization” companion script for A) the bacterium-phage data set and B) the Sphagnum fallax data set. The x-axis shows the available normalization methods within MetaboDirect, while the y-axis shows multiple combinations of the subset methods with different subset parameters. The SPANS score is shown as a color scale with yellow as the highest score. For more information consult the User’s Guide (https://metabodirect.readthedocs.io). Fig. S3. Number of detected peaks that were assigned a molecular formula in the bacterium-phage data set. The sample P_rich_HS2_T30_R4 has few peaks that were assigned a molecular formula and can be a potential outlier during the study. Fig. S4. A) Changes in the molecular class composition of the bacterium-phage exometabolome during the incubation. A reduction in the percentage of lignin-like compounds is observed at 30 minutes after inoculation only for the HS2 phage. B) Violin plot of the changes in the aromatic index reflects the changes in molecular composition, cells infected with the HS2 phage have lower AImod at 30 minutes after inoculation (Tukey HSD test, p-value < 0.05). C) Violin plot showing that double bond equivalence (DBE) of HS2 is reduced after 30 minutes and remains low until the end of the experiment (Tukey HSD test, p-value < 0.05). For B), C) and D) * (p-value < 0.05), ** (p-value < 0.01), *** (p-value < 0.001), **** (p-value < 0.0001). Fig. S5. A) Upset plot showing the number of metabolites that are shared and unique between control and inoculated treatments of the S. fallax leachate. B) Van Krevelen diagram showing metabolites that are shared and unique between control and inoculated treatments of the S. fallax leachate. C) Molecular composition of the unique metabolites showing [file 40168_2023_1476_MOESM3_ESM.docx]

# SUPPLEMENTARY FIGURES


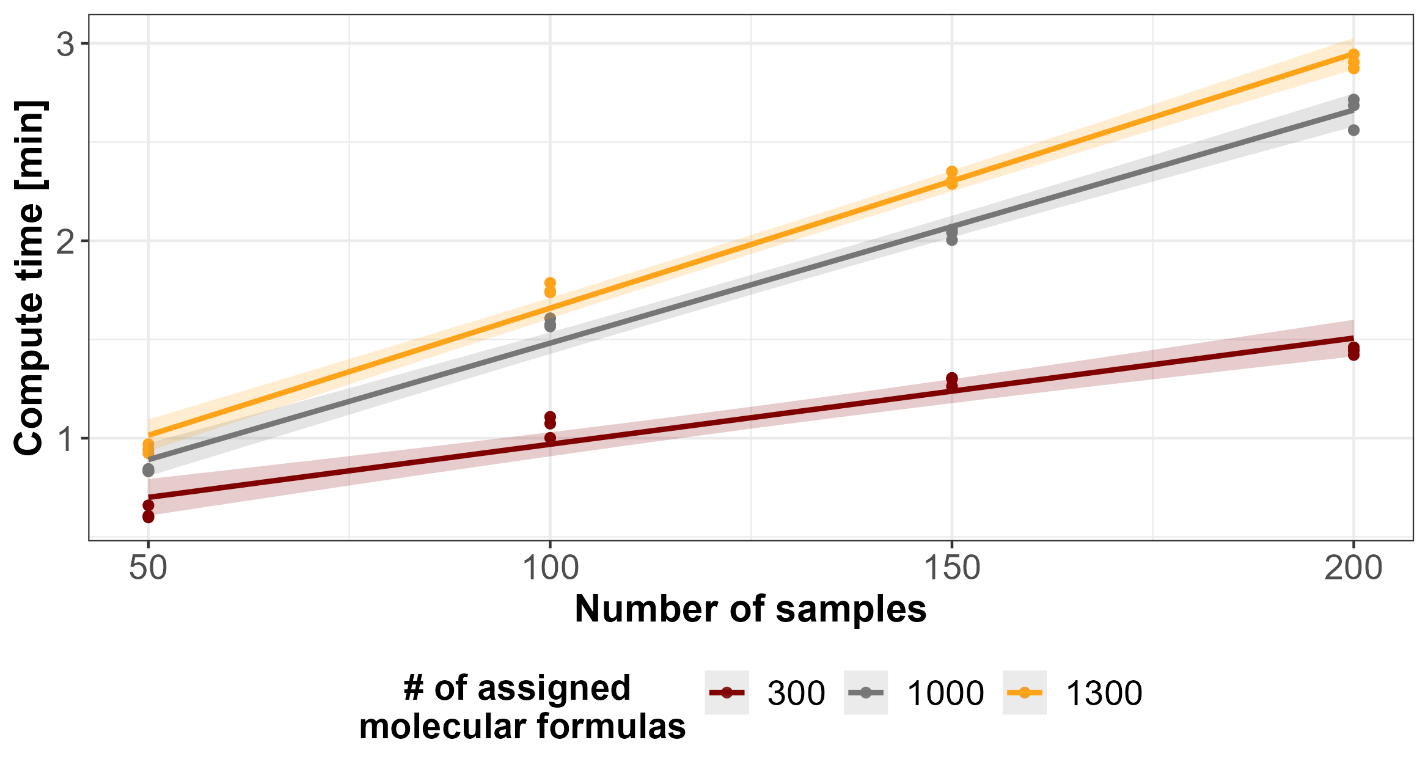


## Supplementary Fig. 1. Compute times of MetaboDirect for data sets with different numbers of samples and different numbers of peaks assigned a molecular formula for any given dataset.


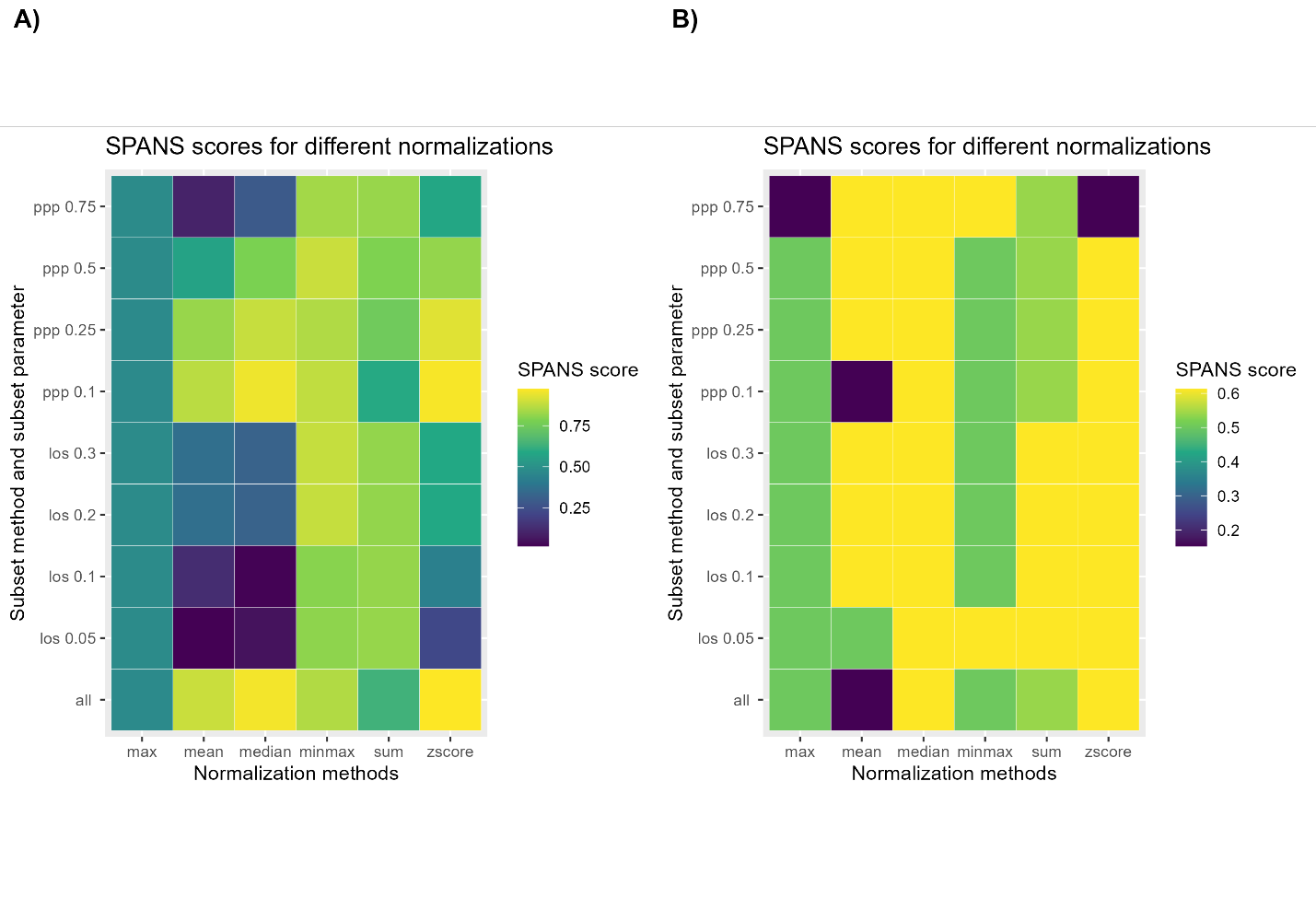


## Supplementary Fig. 2. SPANS score calculated by the “test_normalization” companion script for A) the bacterium-phage data set and B) the *Sphagnum fallax* data set. The x-axis shows the available normalization methods within MetaboDirect, while the y-axis shows multiple combinations of the subset methods with different subset parameters. The SPANS score is shown as a color scale with yellow as the highest score. For more information consult the User’s Guide (https://metabodirect.readthedocs.io).


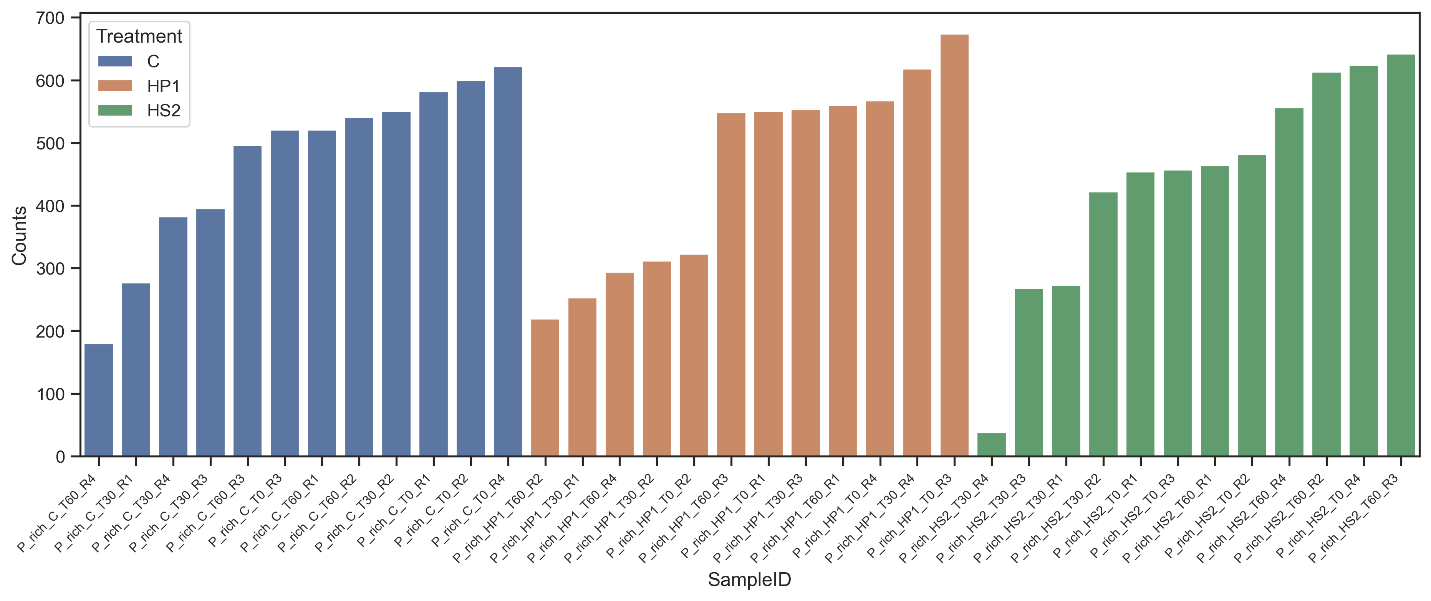


## Supplementary Fig. 3. Number of detected peaks that were assigned a molecular formula in the bacterium-phage dataset. The sample P_rich_HS2_T30_R4 has few peaks that were assigned a molecular formula and can be a potential outlier during the study.


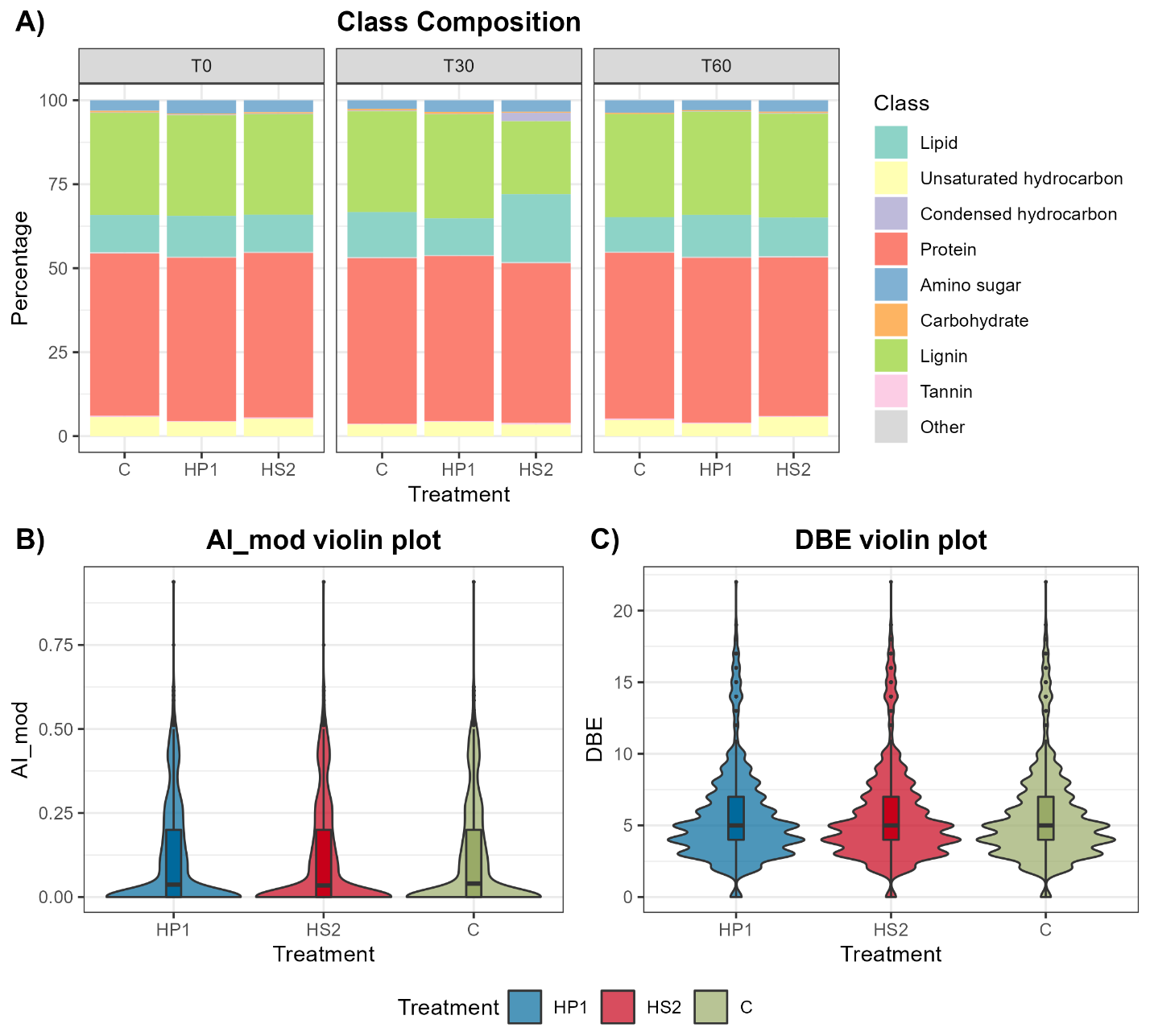


## Supplementary Fig. 4. A) Changes in the molecular class composition of the bacterium-phage exometabolome during the incubation. A reduction in the percentage of lignin-like compounds is observed at 30 minutes after inoculation only for the HS2 phage. B) Violin plot of the changes in the aromatic index reflects the changes in molecular composition, cells infected with the HS2 phage have lower AI_mod_ at 30 minutes after inoculation (Tukey HSD test, p-value < 0.05). C) Violin plot showing that double bond equivalence (DBE) of HS2 is reduced after 30 minutes and remains low until the end of the experiment (Tukey HSD test, p-value < 0.05). For B), C) and D) * (p-value < 0.05), ** (p-value < 0.01), *** (p-value < 0.001), **** (p-value < 0.0001).


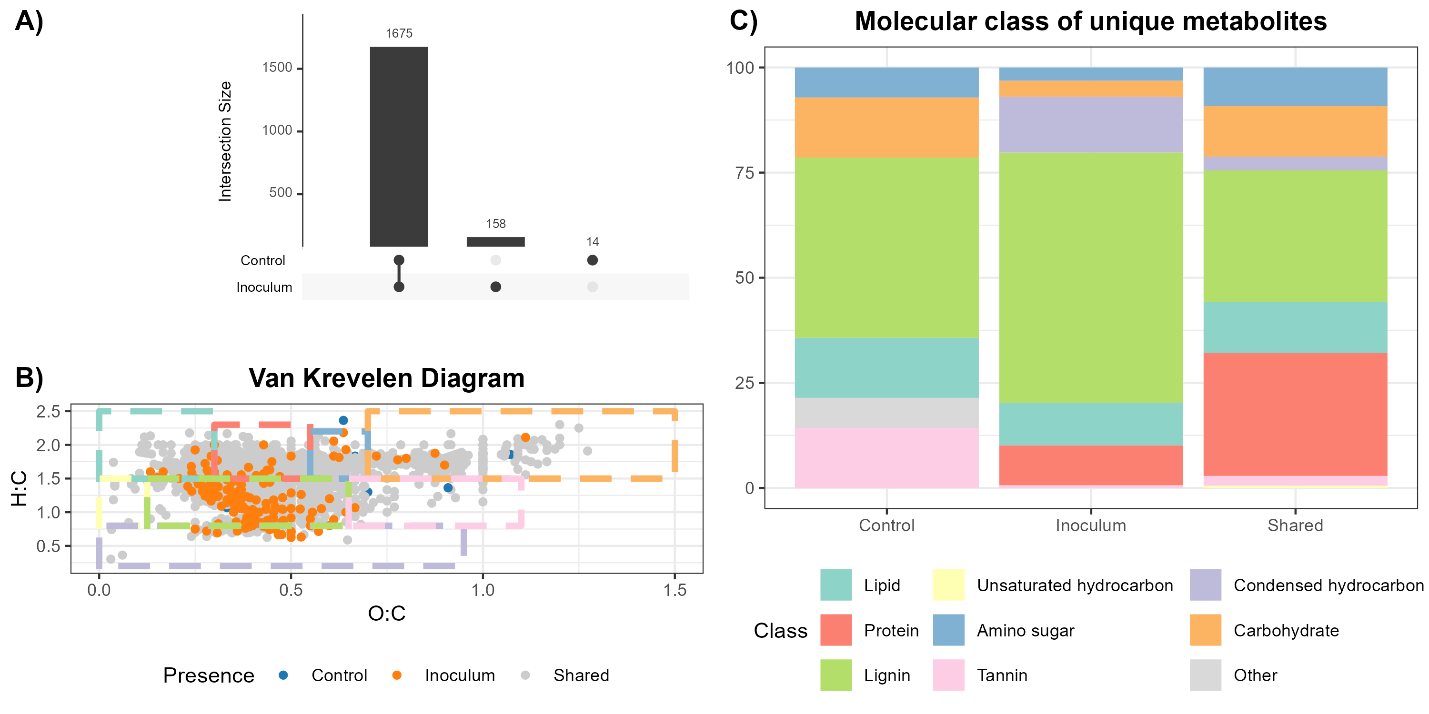


## Supplementary Fig. 5. A) Upset plot showing the number of metabolites that are shared and unique between control and inoculated treatments of the *S. fallax* leachate. B) Van Krevelen diagram showing metabolites that are shared and unique between control and inoculated treatments of the *S. fallax* leachate. C) Molecular composition of the unique metabolites showing that there are unique protein-like, carbohydrate-like, lignin-like and lipid-like metabolites.


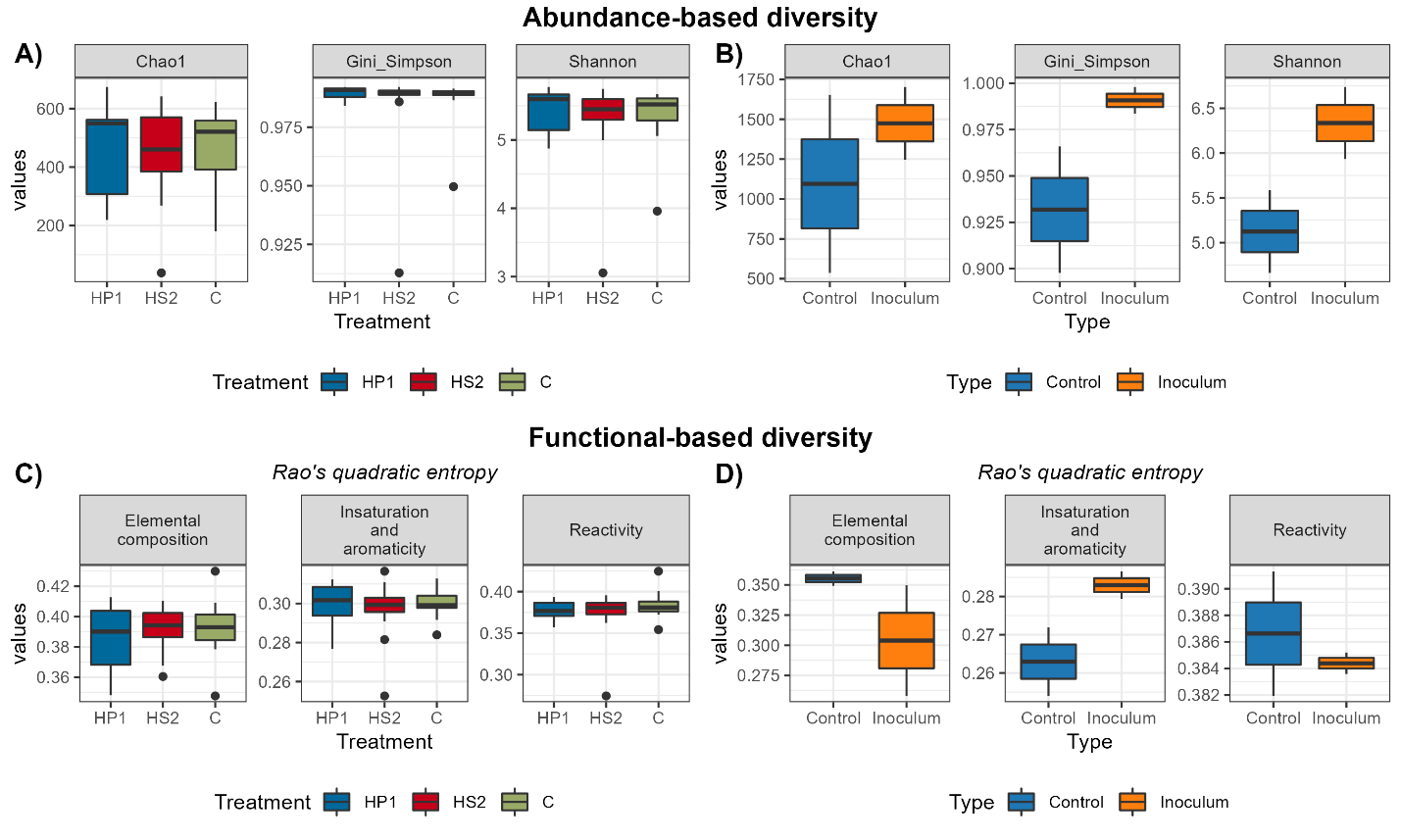


## Supplementary Fig. 6. Results of the chemodiversity analysis of the FT-ICR MS data. A) and B) Abundance-based diversity metrics including the Chao1 richness estimator, Gini-Simpson, and Shannon indexes. C) and D) Functional-based diversity using Rao’s quadratic entropy using different traits: Elemental composition is based on the number of elements in each molecular formula. Insaturation and aromaticity uses DBE and AImod as traits. Reactivity uses Gibbs’ free energy as a trait.


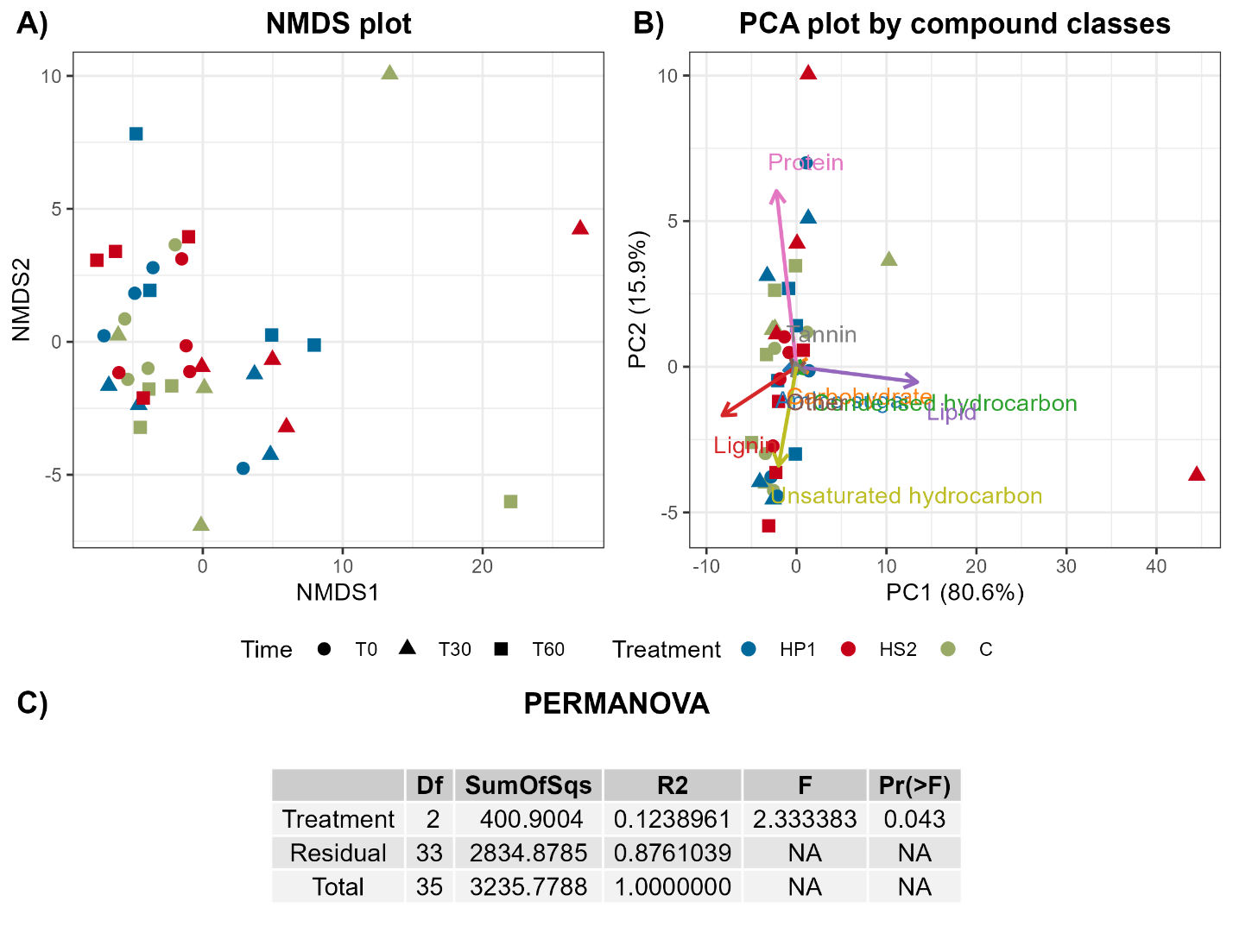


## Supplementary Fig. 7. Multivariate statistical analysis performed by MetaboDirect. A) NMDS plot showed a small clustering of samples based on the content of phosphorus rather than the type of infection, as denoted by the clusters of colored dots B) PCA plots by compound molecular class. Like the NMDS plot, there was no clustering of the sample neither by phage nor time. C) PERMANOVA result, the last column shows the p-value of the analysis. There was not a significant effect of the phage or the time.
